# Supplementary material for: CloneSig can jointly infer intra-tumor heterogeneity and mutational signature activity in bulk tumor sequencing data
Source: Nat Commun. 2021 Sep 9;12:5352. doi: 10.1038/s41467-021-24992-y (PMC8429716; doi:10.1038/s41467-021-24992-y)
Supplement: Supplementary file 2 — Reporting Summary [file 41467_2021_24992_MOESM2_ESM.pdf]

nature research

Corresponding author(s): Jean-Philippe Vert

Last updated by author(s): Jun 22, 2021

Reporting Summary

Nature Research wishes to improve the reproducibility of the work that we publish. This form provides structure for consistency and transparency in reporting. For further information on Nature Research policies, see Authors & Referees and the Editorial Policy Checklist .

Statistics

For all statistical analyses, confirm that the following items are present in the figure legend, table legend, main text, or Methods section.

n/a	Confirmed	
☐	☒	The exact sample size (n) for each experimental group/condition, given as a discrete number and unit of measurement
☐	☒	A statement on whether measurements were taken from distinct samples or whether the same sample was measured repeatedly
☐	☒	The statistical test(s) used AND whether they are one- or two-sided<br>*Only common tests should be described solely by name; describe more complex techniques in the Methods section.*
☐	☒	A description of all covariates tested
☐	☒	A description of any assumptions or corrections, such as tests of normality and adjustment for multiple comparisons
☐	☒	A full description of the statistical parameters including central tendency (e.g. means) or other basic estimates (e.g. regression coefficient) AND variation (e.g. standard deviation) or associated estimates of uncertainty (e.g. confidence intervals)
☐	☒	For null hypothesis testing, the test statistic (e.g. F, t, r) with confidence intervals, effect sizes, degrees of freedom and P value noted<br>*Give P values as exact values whenever suitable.*
☒	☐	For Bayesian analysis, information on the choice of priors and Markov chain Monte Carlo settings
☐	☒	For hierarchical and complex designs, identification of the appropriate level for tests and full reporting of outcomes
☐	☒	Estimates of effect sizes (e.g. Cohen's d, Pearson's r), indicating how they were calculated

Our web collection on statistics for biologists contains articles on many of the points above.

Software and code

Policy information about availability of computer code

Data collection	Data from the TCGA was downloaded using the GDC transfer tool (version v1.4.0), data from the PCAWG, and for the DREAM, PhylogicSim500, SimClone1000 datasets was downloaded directly from the web browser

Data analysis

For analysis, we used
- Python 3.6 (packages pandas==0.24.2, matplotlib==3.0.2, seaborn==0.9.0, scikit-learn==0.20.3 for data manipulation and plots, lifelines==0.22.3 for survival anayses, segment_liftover 0.951 for CNV data preprocessing (partly available on COSMIC, full results obtained from personal communication of Peter Van Loo, using ASCAT 2.4 https://cancer.sanger.ac.uk/cosmic/help/cnv/overview)), and R version 3.3.2

For ITH and signature analyses
- CloneSig (https://github.com/judithabk6/clonesig)
- [PyClone 0.13.0](https://bitbucket.org/aroth85/pyclone/wiki/Installation) (Python 2.7)
- [Sciclone 1.1](https://github.com/genome/sciclone)
- [Tracksig](https://github.com/morrislab/TrackSig)
- [Ccube 205f5e7b89](https://github.com/keyuan/ccube/tree/205f5e7b895fd302019faced3f4acf1a3f15e778)
- [palimpsest](https://github.com/FunGeST/Palimpsest)
- [deconstructSigs 9bbaf15387](https://github.com/raerose01/deconstructSigs/tree/9bbaf15387e1a6221b4437523d12dd950eea80e1)
- [TrackSigFreq 23b2f3f](https://github.com/morrislab/TrackSigFreq/tree/23b2f3f75b344d18d1df6817f3492f8b80047500)
- [DPClust 75f5d7e](https://github.com/Wedge-lab/dpclust/tree/75f5d7ef1e3e53585f86801fde76dd4c4aa86324)
- [PhylogicNDT c229cec](https://github.com/broadinstitute/PhylogicNDT/tree/c229cec570169b6e710e9157c9e102ce37a454cd)

The code for the CloneSig package is available at https://github.com/judithabk6/clonesig, and the code to reproduce all analysis and figures is at https://github.com/judithabk6/Clonesig_analysis

For manuscripts utilizing custom algorithms or software that are central to the research but not yet described in published literature, software must be made available to editors/reviewers. We strongly encourage code deposition in a community repository (e.g. GitHub). See the Nature Research guidelines for submitting code & software for further information.

Data

Policy information about availability of data

All manuscripts must include a data availability statement. This statement should provide the following information, where applicable:
- Accession codes, unique identifiers, or web links for publicly available datasets
- A list of figures that have associated raw data
- A description of any restrictions on data availability

The manuscript includes a data availability statement
Figures A, 7, S36-S68, S103-S108, Table S3 were generated with TCGA data
For the TCGA cohort, we downloaded data from the GDC (Genomic Data Commons) data portal \url{https://portal.gdc.cancer.gov/}. We gathered annotated somatic mutations, both raw variant calling output, whose access is restricted and public mutations (data access project 10569 (dbGaP)), from the unified TCGA pipeline \url{https://docs.gdc.cancer.gov/Data/Bioinformatics_Pipelines/DNA_Seq_Variant_Calling_Pipeline/}, with alignment to the GRCh38 assembly, and variant calling using 4 variant callers: MuSe, Mutect2, VarScan2 and SomaticSniper. Clinical data were downloaded from the cBioPortal~\cite{Gao2013}. Clinical data was downloaded from the CBIO portal at this address http://download.cbioportal.org/${lower_cancer_loc}_tcga.tar.gz with the following values for ${lower_cancer_loc}: acc brca chol dlbc gbm kich kirp lgg luad meso paad prad sarc stad thca ucec uvm blca cesc coad esca hnsc kirc laml lihc lusc ov pcpg read skcm tgct thym ucs. We obtained the TCGA data from the GDC portal at \url{https://portal.gdc.cancer.gov/}. To obtain SNV file list, we used the following query:\texttt {cases.project.program.name in [``TCGA"] and files.data\_type in [``Aggregated Somatic Mutation", ``Masked Somatic Mutation"] and files.experimental\_strategy in [``WXS"]}. ASCAT copy number file list can be obtained using this query \texttt{files.analysis.workflow_type in [``ASCAT2"] and files.data\_type in [``Allele-specific Copy Number Segment"]}. Instructions for download and organization of the files can be found in the companion Github repository (\url{https://github.com/judithabk6/CloneSig_analysis}). To access the restricted part of the dataset, as it is considered potentially identifying, researchers will need to apply to the TCGA Data Access Committee (DAC) via dbGaP (\url{https://dbgap.ncbi.nlm.nih.gov/aa/wga.cgi?page=login}).

Figures 3, 4, 6 were generated using the PhylogicSim500 dataset available at https://data.mendeley.com/datasets/ by4gbgr9gd/1.
Figures 3, S34 and S35 were generated using the SimClone1000 dataset available at https://data.mendeley.com/datasets/ by4gbgr9gd/1.
Figures 3, and S28 to S33 were generated using the DREAM dataset available at at Synapse storage https: //www.synapse.org/#!Synapse:syn2813581/files/
Figures S69 to S104 were generated with PCAWG data
PCAWG mutations were downloaded from the ICGC data portal at https://dcc.icgc.org/releases/ PCAWG/consensus_snv_indel, CNV data at https://dcc.icgc.org/releases/PCAWG/consensus_cnv (mirrored on the Synapse platform (https://www.synapse.org/, with accession number syn8042988 for CNA data, syn8272483 for purity and ploidy and syn7364923 for SNV). Access for the restricted files (potentially identifying information) can be granted upon application to the TCGA Data Access Committee (DAC) via dbGaP (https://dbgap.ncbi.nlm.nih.gov/aa/wga.cgi?page=login) for access to the TCGA portion of the dataset, and to the ICGC Data Access Compliance Office (DACO; http://icgc.org/daco) for the ICGC portion.

Field-specific reporting

Please select the one below that is the best fit for your research. If you are not sure, read the appropriate sections before making your selection.

☒ Life sciences ☐ Behavioural & social sciences ☐ Ecological, evolutionary & environmental sciences

For a reference copy of the document with all sections, see nature.com/documents/nr-reporting-summary-flat.pdf

Life sciences study design

All studies must disclose on these points even when the disclosure is negative.

Sample size	For the simulation studies to assess the performance of CloneSig, a high number of parameters vary (set of active signatures, number of clones, number of mutations, CNV abundance), so one simulation per combination was sufficient to analyse one parameter at a time (for each of the 6 tested numbers of clones, there are 1302 repeats with varying combinations of 31 signature sets, 6 CNV abundances and 7 number of SNVs etc). This yielded performance estimates with very small 95% confidence interval estimates obtained by bootstrapping, compared to the effect we analyze. A second set of 6300 simulated samples was generated with no signature variation between clones.<br><br>A third set of 12000 simulated samples was generated to assess the separating power of CloneSig, with the same rationale of 1 repeat per combination of all parameters (number of SNVs, sequencing depth, CCF distance between clones, cosine distance between mutation types profiles).<br><br>Finally, a last simulated dataset of 2772 samples was generated to evaluate the sensitivity of the statistical test to assess whether the change of signature activities between clones is significant, with varying number of clones, SNVs, sequencing depth, CNV abundance, number of clones, and minimal cosine distance between clones.<br><br>In those cases, the number of simulations was large enough to obtain sufficiently small 95% confidence intervals (assessed by bootstrap) in order to detect the significant effects that we discuss in the manuscript.<br><br>We additionally included three external simulated datasets to the evaluation: the DREAM dataset, with 6 tumors, at 5 different sequencing depths, the PhylogicSim500 with 500 samples, and the SimClone1000 dataset with 972 samples.<br><br>Regarding experiments on the TCGA and the PCAWG cohorts, all available patients were considered.<br><br>We did not control the number of samples in the external simulated and real data sets, and only report significant findings when supported by statistical tests.
Data exclusions	For the TCGA, we analyzed patients with available public and protected mutation data, CNV data, and clinical data. We excluded patients for which one of those data type was not available, or that were mentioned in the annotation files provided with mutation data, or that had non-positive survival times. In the case where several samples were available for one patient, we kept the one corresponding to the primary tumor. All patient selection code is available here https://github.com/judithabk6/Clonesig_analysis/blob/master/signature_code/get_patient_list.py<br><br>No data was excluded from the generated simulations.<br>For the PhylogicSim500 and the SimClone1000 datasets, samples with more than 10,000 SNVs were excluded for computation time concerns
Replication	CloneSig produces the same results when run with the same starting conditions (including the random seed for initialization).<br><br>Regarding results on the TCGA and PCAWG, a careful comparison with similar findings on a distinct WGS cohort is reported, and complete results are reported (graphically in the Supplementary). To foster future comparison on the same samples with potentially future methods or cohorts, all results on those cohorts are available at https://github.com/judithabk6/Clonesig_analysis/tree/master/result_tables.
Randomization	not applicable. No randomization was required in our experimental setting.
Blinding	not applicable. No blinding was required in our experimental setting.

Reporting for specific materials, systems and methods

We require information from authors about some types of materials, experimental systems and methods used in many studies. Here, indicate whether each material, system or method listed is relevant to your study. If you are not sure if a list item applies to your research, read the appropriate section before selecting a response.

Materials & experimental systems

n/a	Involved in the study
☒	☐ Antibodies
☒	☐ Eukaryotic cell lines
☒	☐ Palaeontology
☒	☐ Animals and other organisms
☒	☐ Human research participants
☒	☐ Clinical data

Methods

n/a	Involved in the study
☒	☐ ChIP-seq
☒	☐ Flow cytometry
☒	☐ MRI-based neuroimaging
